# Correlates of adherence to antipsychotic Medications in Patients with Schizophrenia and Bipolar Disorders

**DOI:** 10.1192/j.eurpsy.2025.2282

**Published:** 2025-08-26

**Authors:** J. Zaouali, F. Askri, S. Ellini, M. Cheour, R. Damak, S. Hallit, F. Fekih-Romdhane

**Affiliations:** 1 Psychiatry, Razi Hospital, Tunis, Tunisia; 2 Research, School of Medicine and Medical Sciences, Holy Spirit University of Kaslik, P.O. Box 446, Jounieh, Kaslik, Lebanon; 3 Psychiatry, Tunis El manar university, faculty of Medicine of Tunis, Tunis, Tunisia

## Abstract

**Introduction:**

The World Health Organization defines non-adherence to treatment as “a situation where an individual’s medication-taking behavior diverges from the agreed recommendations of healthcare professionals”. Individuals with psychiatric disorders frequently encounter challenges in adhering to their prescribed treatments, which is due amon other factors to a lack of insight. The non-adherence can lead to increased relapse rates, diminished treatment efficacy over time, and adverse effects on both the individual and the broader community.

**Objectives:**

This study’s objectives were towfold : (1) to investigate medication adherence among patients with schizophrenia and bipolar disorders under antipsychotics, and (2) to identify various factors associated with non-adherence to antipsychotic treatments.

**Methods:**

A cross-sectional study was conducted at Razi Hospital, Tunisia, between December 2023 and January 2024. Were included patients who attended the outpatient clinic during the study period, who were in remission for at least one month and who were receiving antipsychotic medication. The Brief Adherence Rating Scale, the Positive and Negative Syndrome Scale and the Glasgow Antipsychotic Side-effects Scale were administered to all patients.

**Results:**

The study included 35 male patients with a mean age of 39 years ± 11 years. Schizophrenia was diagnosed in 86% (N=30) of the participants. Of these, 49% were prescribed first-generation antipsychotics (N=17), while 51% (N=18) were prescribed second-generation antipsychotics. More than half of the patients (63%) demonstrated non-adherence to their treatment regimen. Among these, 65% exhibited moderate to severe lack of insight into their illness, and 66% had not received psychoeducation about their condition. A significant association was found between non-adherence and moderate to severe lack of insight (p=0.000, OR=4, 95% CI [1.4-10]), lack of psychoeducation for the patient (p=0.02, OR=2, 95% CI [1-4.3]), and lack of psychoeducation for the caregiver (p=0.05, OR=1.7, 95% CI [0.9-3.2]). Binary logistic regression analysis indicated that lack of insight (p=0.01, OR=3.6) remained a significant risk factor for non-adherence.

**Conclusions:**

This study underscores significant association between lack of insight and non-adherence to antipsychotic medications. Enhancing insight through early psychoeducational interventions could potentially improve medication adherence and positively influence long-term clinical and functional outcomes for patients.

**Disclosure of Interest:**

None Declared

